# Engineering of the Embryonic and Adult Stem Cell Niches

**DOI:** 10.5812/ircmj.7541

**Published:** 2013-02-05

**Authors:** Mohsen Hosseinkhani, Reza Shirazi, Farzad Rajaei, Masoud Mahmoudi, Navid Mohammadi, Mahnaz Abbasi

**Affiliations:** 1 Department of Anatomy, Qazvin University of Medical Science, Qazvin, IR Iran; 2 Department of Community Medicine, Tehran University of Medical Science, Tehran, IR Iran; 3 Department of Rheumatology, Qazvin University of Medical Science, Qazvin, IR Iran

**Keywords:** Stem Cells, Biocompatible Materials, Cellular Microenvironment, Cell Differentiation

## Abstract

**Context:**

Stem cells have the potential to generate a renewable source of cells for regenerative medicine due to their ability to self-renew and differentiate to various functional cell types of the adult organism. The extracellular microenvironment plays a pivotal role in controlling stem cell fate responses. Therefore, identification of appropriate environmental stimuli that supports cellular proliferation and lineage-specific differentiation is critical for the clinical application of the stem cell therapies.

**Evidence Acquisition:**

Traditional methods for stem cells culture offer limited manipulation and control of the extracellular microenvironment. Micro engineering approaches are emerging as powerful tools to control stem cell-microenvironment interactions and for performing high-throughput stem cell experiments.

**Results:**

In this review, we provided an overview of the application of technologies such as surface micropatterning, microfluidics, and engineered biomaterials for directing stem cell behavior and determining the molecular cues that regulate cell fate decisions.

**Conclusions:**

Stem cells have enormous potential for therapeutic and pharmaceutical applications, because they can give rise to various cell types. Despite their therapeutic potential, many challenges, including the lack of control of the stem cell microenvironment remain. Thus, a greater understanding of stem cell biology that can be used to expand and differentiate embryonic and adult stem cells in a directed manner offers great potential for tissue repair and regenerative medicine.

## 1. Context

Stem cells are primitive cells found in many multi-cellular organisms. Self-renewal and potency are two defining properties of stem cells. Self-renewal is the ability to perform numerous cell cycle divisions, each resulting in two identical daughter cells, while differentiation potency defines the differentiation capability of stem cells into mature cell types. The two main categories of mammalian stem cells are: embryonic stem (ES) cells, which are derived from blastocysts, and adult stem cells, which are found in adult tissues. ES cells have the potential to differentiate into all mature cell types except extra embryonic tissue (1-3). ES cell differentiation can be “ex vivo” induced from cell aggregates, called embryonic bodies (EBs), which initiate many developmental processes and generate derivatives of the three primary germ layers (ectoderm, mesoderm, and endoderm) (4-6). Because of their ability to differentiate into all the cell types of an adult organism, ES cells are useful for cell-replacement therapies (7-9) for a number of diseases including Alzheimer’s disease, Parkinson’s disease, spinal cord injury, heart disease, and diabetes (10-14). To overcome the ethical controversy regarding the derivation of ES cells, recent studies have developed other methods of driving stem cells that exhibit ES cell-like properties. For example, induced pluripotent stem (iPS) cells are reprogrammed mature cell from various sources including fetal and neonatal, as well as cell isolated from skin biopsies of adult tissues (15). Pluripotency of iPS cells is comparable to ES cells upon analysis by using teratoma formation and in vitro differentiation assays (16-19). Although the developmental potential of iPS cells has not been clearly determined, the generation of these cells through direct reprogramming has the potential to generate personalizable stem cells without the use of embryos. Adult stem cells are another class of stem cells comprised of undifferentiated cells found in many tissues of an adult organism. They have an extensive self-renewal capability and the ability to differentiate into various specialized cell types (i.e. blood, muscle, and nerve cells) (20, 21). The primary roles of adult stem cells in a living organism are to maintain and repair tissues. Although in most systems, they give rise to cells of the tissues from which they are derived, adult stem cells may have the ability to differentiate across the germ layers into cells of other tissues (22, 23). Adult stem cells are a particularly promising cell type, because they are easy to obtain, less controversial, and, if obtained from autologously, are less immunogenic than ES cells (24, 25). However there are disadvantages to the use of adult stem cells which include limited differentiation potential as well as difficulties in their isolation and expansion in vitro (26). Despite the therapeutic and pharmaceutical significance of embryonic and adult stem cells, a significant challenge to their widespread clinical use is to control their self-renewal and differentiation to desired cell types. Although conventional methods for culturing stem cells have greatly enhanced our understanding of stem cell behavior, they have limitations on the spatial and temporal regulation of stem cell microenvironments (i.e. stem cell niches) that can be generated in culture. In addition, these methods are not easily adapted to high-throughput experimentations that aim to examine the effects of various signals within the stem cell microenvironment. Recently, micro scale technologies are emerging as powerful tools to control the cellular microenvironments (27, 28). These technologies, which have been adapted from the microelectronics industry, can be used to study the spatial and temporal effects of cell-cell, cell-matrix, and cell-soluble factor interactions (29). Using techniques such as surface micro patterning, high-throughput microarrays, microfluidics, and three-dimensional (3D) micro engineered scaffolds, various aspects of the cellular microenvironment can be controlled in a manner that is usable to high-throughput studies.

## 2. Evidence Acquisition

Here, we provide a brief overview of the applications of micro scale technologies and their applications for regulating the behavior of stem cells.

### 2.1. Micro engineering the Embryonic Stem Cell Niche in Vitro

ES cell fates (i.e. self-renewal, proliferation, differentiation, migration, and apoptosis) are regulated by both intrinsic and extrinsic factors (30, 31). To enable precise control of the ES cell niche, it is necessary to control cell-cell contact, cell-extracellular matrix (ECM) interactions, cell-soluble factor interactions, as well as mechanical and electrical stimuli in a temporally and spatially regulated manner (27). A number of engineering approaches such as surface patterning, high-throughput microarrays, microfluidics, and 3D scaffolds have been applied to control the ES cell niche.

### 2.2. Surface Patterning for Culturing ES Cells

**Figure 1:**
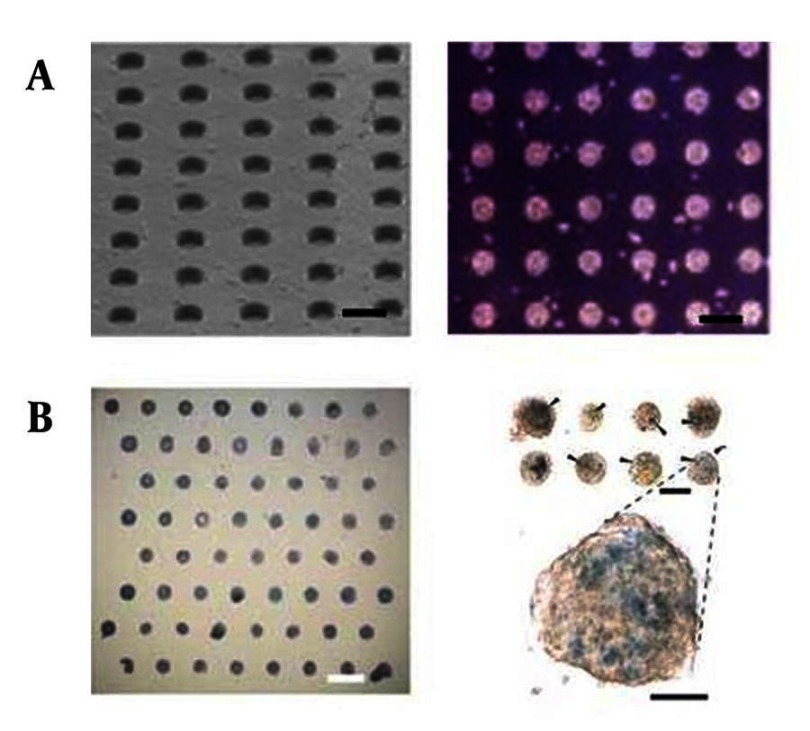
Microarrays for Studying ES Cell Behavior A) Scanning Electron Microscopy image of a PEG micro well array (left) and light microscopy image of ES cells cultured within a micro fabricated PEG micro well array of 75 μm diameter for 10 days (right) (36). The size and shape of EBs were controlled within an array of PEG micro wells. Scale bars are 200 µm; B) ECM microarray for studying ES cell differentiation (left) and bright-field micrograph of X-gal−stained ECM microarray conditions after 3 days of culture in retinoic acid (right) (88). The ECM array contained a combination of collagen I, III, laminin, and fibronectin. The scale bars are 1mm (left) and 250 µm (right).

The proliferation and differentiation of ES cells can be controlled by regulating homotypic (contact with the same cell type) and heterotypic (contact with other cell types) cell-cell contact. These interactions can be manipulated by patterning cells within geometrically defined adhesive regions on two-dimensional (2D) surfaces (28, 32, 33). To pattern ES cells on surfaces, a number of techniques have been used such as micro-fabricated stencils (34), micro-contact printing (35), micro-topographies (36, 37), and micro-patterned layer-by-layer deposition of ionic biomaterials (38). In most cases, these technologies have been used to study ES cell fates by controlling ES cell colony formation. For example, to regulate homotypic cell-cell contacts, micro-fabricated adhesive stencils were used to pattern mouse ES cells (34). Within these cultures, the size of the ES cell-aggregates generated within micro-patterns directed stem cell differentiation. Small cell aggregates (100 µm diameter) enhanced ectoderm differentiation, while large cell aggregates (500 µm diameters) induced a higher frequency of mesoderm and endoderm differentiation. Micro-contact printing has also been used to regulate human ES cell fates. Micro-contact printed substrates (200-800 µm diameters) that generated islands of ES cells were shown to regulate the self-renewal of human ES cells by local modulation of pSmad1 agonists and antagonists (35). These experiments have revealed that the levels of Smad1 antagonists were increased with decreasing pluripotency in smaller colonies. Therefore, by modulating the degree of homotypic cell-cell interactions, surface patterning methods could be useful for regulating cell fate decisions and understanding their underlying biology. Although micro-fabricated stencils and micro-contact printed substrates are useful for cell patterning, they are often limited to adhesive cells and 2D monolayers. To address this limitation, micro-well arrays of cell-repellent poly (ethylene glycol) (PEG) hydrogels were developed to control the formation of EBs (Figure 1) (36). EBs mimics the structures of the developing embryos and gives rise to a wider spectrum of cell types. Conventional ES cell culture systems such as hanging drop and suspension culture cannot be easily manipulated to generate homogeneous cell aggregates in a highly scalable manner. This is because suspension cultures generate heterogeneous EBs, while hanging drop cultures cannot be easily scaled-up. In contrast, PEG hydrogel micro-wells could be used to control the size and shape of ES cell aggregates and result in the generation of EBs with uniform sizes and shapes. Experiments have demonstrated that cells that were cultured within PEG micro-wells for 10 days remained viable (> 95%) and resulted in a more uniform differentiation response (36). Although the first generation version of the micro-well arrays (36) could be used to control EB size and shape, it was difficult to obtain homogeneous EBs with high retrieval efficiency. To overcome this limitation as well as to characterize and optimize protein and cell repellent properties of PEG micro-well arrays, PEG macromeres with different molecular weights were examined (37). It was demonstrated that PEG hydrogels with higher molecular weights were more resistant to protein and cell adhesion and generated more homogeneous EBs. Moreover, a micro-fabricated polymer chip made by using elastomeric stencils has been developed to study ES cell contact (39). In this approach, murine ES cells were immobilized within micro-wells as either individuals or clusters to demonstrate that cell-cell interactions significantly decreased the ES cell colony formation. Also, other studies have used a micro-well-based method to demonstrate that human ES cells can be maintained in an undifferentiated form for several weeks in culture within micro-wells with an adhesive coating (40). To control heterotypic cell-cell contacts, a number of patterned co-culture approaches have been explored. For example, ES cells and fibroblasts were co-cultured via layer-by-layer deposition of hyaluronic acid and poly-L-lysine (38). In this approach, a glass substrate was initially patterned with negatively charged hyaluronic acid and fibronectin. Fibronectin patterns, which were initially more adhesive, resulted in patterned deposition of cells. Non-biofouling hyaluronic acid patterns were subsequently switched to cell adhesive substrates by adsorption of positively charged poly-L-lysine or collagen for attaching the second cell type. We have recently demonstrated the merger of micro fabrication, and biomaterials technologies to control the cluster size and the numbers of human ES cells co-cultured with murine embryonic fibroblasts (MEFs) (41). In this approach, polymeric micro wells were used to control the size and uniformity of hES cell clusters in co-culture with MEFs and to produce homogeneous cell aggregates for differentiation experiments.

Micro-fabricated parylene-C stencils were also used to pattern and immobilize ES cells (42) as well as to generate patterned co-cultures with dynamic control of heterotypic cell-cell interactions (43). Furthermore, reversibly sealable parylene membranes were used to generate dynamic patterned co-cultures. Using this approach, ES cells were co-cultured with fibroblasts and hepatocytes in a spatially and temporally regulated manner. Therefore, ES cells were initially co-cultured with fibroblasts that were seeded on the stencil. Removal of the stencil was used to remove the fibroblasts and to enable the sequential deposition of hepatocytes (43). Although much more in depth analysis has yet to be performed using these systems, it appears that surface patterning techniques are useful for studying cell-cell contacts and controlling the size and shape of ES cell aggregates.

### 2.3. Microarrays and Microfluidics for Controlling Stem Cell Behavior

Micro scale technologies are enabling tools for directing stem cell differentiation and studying cell-matrix interactions, because they are able to generate miniaturized microarrays that can be used to perform high-throughput experiments. For example, a high-throughput polymer microarray was fabricated by using robotic spotters for analyzing various ES cell-biomaterial interactions (44). Within this synthetic polymer array, 1700 human ES cell-biomaterial interactions were simultaneously characterized for stem cell growth and differentiation. Similar to synthetic biomaterial arrays, an ECM microarray was also developed to study stem cell behavior (Figure 1) (45). This platform, which consisted of multiple combinations of the various ECMs (i.e. laminin, fibronectin, collagen I, collagen III, and collagen IV), was used for analyzing the differentiation of mouse ES cells into hepatocytes. Using this platform, it was demonstrated that collagen I and fibronectin induced a higher degree of differentiation of ES cells into early hepatic fates. Therefore, by using microarrays a number of cell-biomaterial interactions could be analyzed for obtaining optimized conditions and discovering novel interactions that were previously difficult to determine. Although ES cells are a promising cell source for regenerative medicine, the lack of optimized conditions to direct their differentiation limits their promise (46). Currently, large combinatorial studies with multiple combinations and concentrations of various growth factors are expensive and time consuming by using conventional culture methods. Microfluidic platforms may be used to analyze ES cell-microenvironment interactions in a manner that is usable to high-throughput screenings. For example, a multi-phenotype cell microarray was incorporated in an array of reversibly sealed poly (dimethylsiloxane) (PDMS) microfluidic channels to enable the testing of multiple chemical factors on arrays of different cell types (including ES cells) (47). This device could be useful for high-throughput drug screening and cell-based diagnostic assays. Microfluidic devices that can generate homogeneous EBs have also been generated (48).These microfluidic devices consisted of two micro channels separated by a semi-porous polycarbonate membrane (5 µm pore sizes) that was resistant to cell adhesion. The upper micro channels facilitated cell capture to form cell aggregates and the lower micro channels enabled continuous media perfusion. It was demonstrated that EBs that were harvested from this microfluidic device differentiated into neuronal cells. Thus, microarrays and microfluidics could be useful tools to manipulate cell-microenvironment interactions in a controlled manner. Future research in this area will focus on more widespread use of this technology for various types of biological systems as well as overcoming the challenges associated with current systems such as lack of desired robustness and the need for extensive expertise and specialized equipment.

### 2.4. 3D Scaffolds for Culturing Es Cells 

In vivo, cells are embedded in a 3D complex matrix with a well-defined geometry, which provides physical and chemical support and mediates the exchange of soluble nutrients and waste. To mimic living tissues, 3D scaffolds have been widely used in tissue engineering applications to provide cells with a suitable growth environment in vitro, with optimal oxygen levels, effective nutrient transport as well as physical and chemical cues (49-51). Micro-fabricated 3D scaffolds can be used to generate porous scaffolds for generating 3D tissue constructs (52-56). Previous research has characterized the convective and diffusive mass transfer of solutes (5, 53), studied cell migration (52, 57), and analyzed adult stem cell behavior within micro-fabricated tissue scaffolds. However, to our knowledge no study so far has analyzed the behavior of ES cells within micro-fabricated 3D scaffolds, even though the seeding of ES cells on conventional biodegradable scaffolds has shown to be a promising approach for tissue engineering (58). Here, we review the application of conventional 3D scaffolds for ES cell cultures. Biodegradable scaffolds have been used in combination with soluble factors to direct stem cell differentiation into different lineages. For example, human ES cells were seeded on 3D scaffolds fabricated from poly(lactic-co-glycolic acid) (PLGA)/poly(L-lactic acid) (PLLA) (58) and treated with various growth factors (i.e. retinoic acid, active-A, insulin-like growth factor (IGF), and transforming growth factor (TGF-β)) to direct multi-lineage differentiation. Within these scaffolds, active-A and IGF were shown to induce endodermal differentiation, while retinoic acid enhanced ectodermal differentiation (including neuroectodermal lineages). TGF-β was found to increase the gene expression of cartilage matrix proteins. In another example, biocompatible fibrin scaffolds were used to direct the differentiation of murine ES cells into neuronal lineages (59). Mouse ES cells exposed to retinoic acid exhibited enhanced proliferation and differentiation into neural progenitors when cultured on 3D fibrin scaffolds than on 2D substrates. Both the physical and chemical properties of the 3D scaffolds have been shown to significantly improve the differentiation efficiency of ES cells. For example, to investigate the effects of physical properties (i.e. pore size, polymer concentration and compression modulus) of 3D scaffolds on the differentiation efficiency of ES cells, PLLA scaffolds with various polymer concentrations and pore sizes were fabricated (60). It was found that 3D scaffolds with smaller pore sizes, higher polymer concentrations, and higher cell seeding densities induced hematopoietic differentiation of ES cells. Although further studies are required to clarify the effects of these parameters on other stem cell fate decisions, this area of research is promising for generating 3D tissue constructs. In addition to physical properties, chemical compositions of the 3D scaffolds are also important in ES cell growth and differentiation (61). For example, EBs cultured within semi-interpenetrating polymeric networks (SIPN) containing collagen, fibronectin, and laminin differentiated differently based on the chemical composition of the scaffolds. High collagen concentrations were necessary to induce EB differentiation and inhibit apoptosis, high fibronectin concentrations in collagen scaffolds resulted in increased endothelial differentiation, and high laminin concentrations resulted in the formation of cardiomyocytes (61). Large scale cell expansion is vital for the success of clinical applications of ES cells. Conventional ES cell expansion is carried out in ECM-coated 2D substrates, which is limited by surface area. 3D scaffolds can potentially be used to generate a larger amount of ES cells by providing larger space for the self-renewal of ES cells. For example, murine ES cells cultured in 3D polyethylene terephthalate (61) fibrous matrices were maintained in an undifferentiated state and proliferated more than ES cell that were cultured on 2D substrates (62). The matrix pore size was shown to significantly influence ES cell expansion with smaller pore (30-60 µm) matrices resulting in a higher proliferation rate. Furthermore, oxygen depletion was proved to be the main reason for poor cell proliferation in long-term cultures, which could be improved by perfusion culture (62). 3D scaffolds incorporated with ES cell-derived endothelial cells have also been shown as a promising approach to address a major challenge of engineering tissues, namely the lack of proper vascularization (63). Oxygen and other nutrients can only diffuse a short distance before being consumed (a few hundred micrometers at most). Without an intrinsic vascularized network, the maximal thickness of engineered tissue is approximately 150-200 µm because of oxygen diffusion limitations (64). Directed differentiation of ES cells into endothelial cells within the 3D scaffolds may be useful for generating vascularized tissue constructs without diffusion limitation. For instance, ES cells that were cultured in 3D collagen gel constructs differentiated into endothelial cells, which formed 3D vascular structures (65). Within two weeks, vessel-like structures were generated with a number of endothelium and multicellular lumenal organizations. Therefore, engineered 3D scaffolds that provide physical and biochemical support and contain vascularized structures may be useful in directing cell fates in a controlled manner.

### 2.5. Micro engineering the Adult Stem Cell Niche in Vitro

Adult stem cells are an attractive cell source for regenerative medicine. Despite their significance, adult stem cells isolated from adult tissues exhibit limited proliferation capacity in vitro (66). The limited proliferation ability of adult stem cells makes it difficult to expand sufficient cells in conventional cultures for therapeutic applications (67). To address the limitations imposed by conventional cultures, engineered microenvironments can be used to regulate cell-cell contact, cell-ECM, cell-soluble factor, and cell-mechanical stimuli interactions in a controlled manner. Here, we describe the use of micro engineering approaches such as surface patterning, microfluidics, and 3D scaffolds for altering adult stem cell behavior.

### 2.6. Surface Patterning, Microarrays, and Microfluidics for Studying Adult Stem Cells

**Figure 2.**
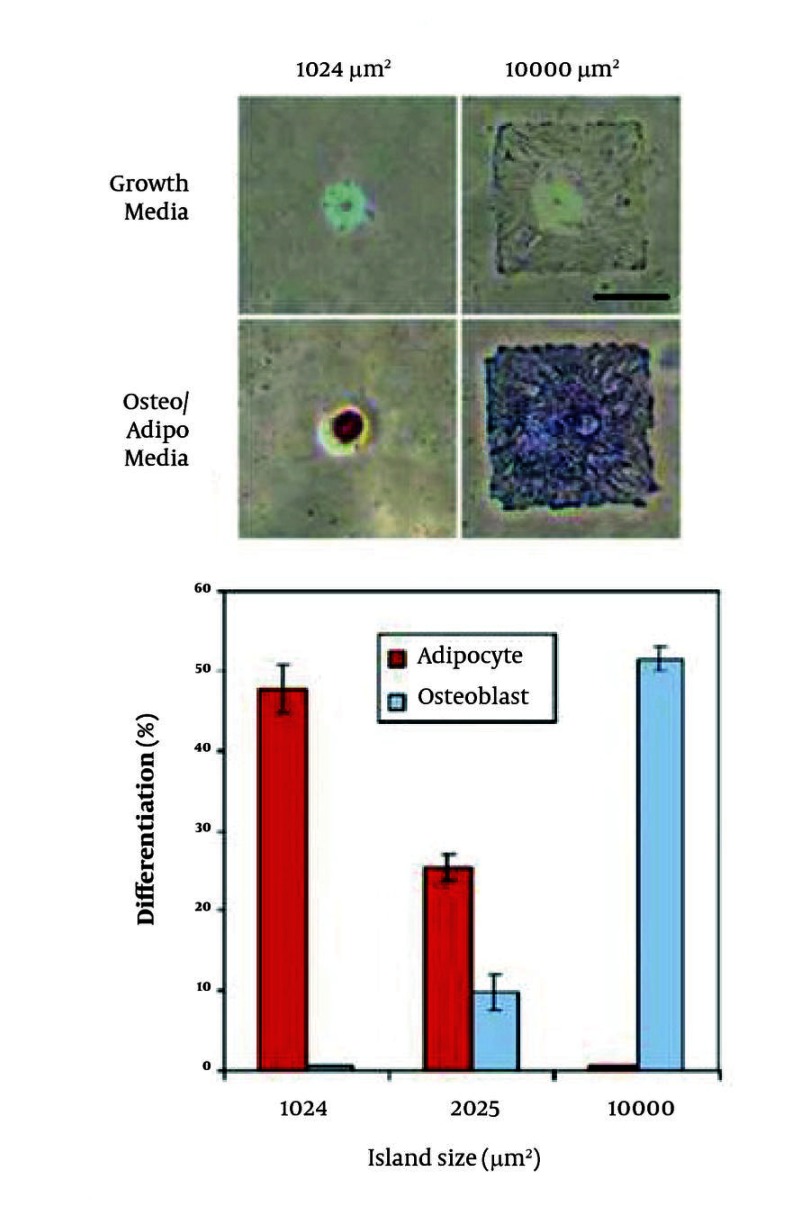
Human MSC Differentiation on Micro patterned Substrates A) Cells cultured on small (1,024 µm2) and large (10,000 µm2) islands differentiated into adipocytes and osteoblasts respectively after 1 week. Lipids stain red and alkaline phosphatase stains blue. Scale bar is 50 µm; B) Differentiation efficiency of hMSCs plated onto 1024, 2025, and 10,000 μm2 islands after 1 week of culture in mixed media without aphidicolin.

Cell patterning on 2D surfaces is important for directing adult stem cell fates (27), because cellular micro patterning can be used to control the spatial arrangement of cells relative to each other. Furthermore, cell patterning can be used to regulate the cell shape and the resulting cytoskeletal structure to direct adult stem cell fate decisions. As an example, the differentiation of human mesenchymal stem cells (MSCs) was controlled by micro patterned substrates (Figure 2) (68). It was demonstrated that human MSCs cultured on small islands (1,024 µm2) differentiated into adipocytes, while osteogenesis of human MSCs occurred on large islands (10,000 µm2). Cell-compatible, biomaterial microarrays fabricated by robotic spotters have the potential to control stem cell behavior in a high-throughput manner (69). Human MSCs were cultured on 24 polymers of different composition and molecular weight. The cell attachment and growth were studied on 3456 individual polymer spots. Thus, a polymeric microarray enabled the high-throughput screening of human MSCs-biomaterial composite interactions. Biomaterials can also significantly affect the proliferation and apoptosis of stem cells. A grid-based platform was used to assess stem cell-biomaterial interactions (70). One hundred and forty combinations of 7 stem cell types and 19 different polymers enabled the systematic screening of cell-biomaterial combinations. The material topography, cell adhesion, proliferation, cytotoxicity, and apoptosis were characterized by using this multiplex assay. Cell adhesion, proliferation, and apoptosis were supported or inhibited by different polymers. Therefore, the study of stem cell-biomaterial interaction is important for stem cell-based tissue engineering and cell-based therapies. Microfluidic devices can be used to manipulate cell-soluble factor interactions by generating temporally and spatially distinct regions by using laminar flow of fluids (71-74). For example, human neural stem cells were cultured in a gradient-generating microfluidic device and exposed to concentration gradients of growth factor mixtures containing epidermal growth factor (EGF), fibroblast growth factor (FGF), and platelet-derived growth factor (PDGF) (71). These experiments indicated that cells exposed to higher concentrations of growth factors exhibited less differentiation and cells exposed to lower concentrations of growth factors differentiated into astrocytes. Micro fabricated platforms were also used to track individual cells to study proliferation and differentiation of adult hippocampal progenitor cells (75). The proliferation dynamics of heterogenous neural stem cell populations were investigated by using this micro fabricated platform to clonally analyze the effects of retinoic acid and forskolin on neuronal differentiation. In addition, an automated microfluidic cell culture screening device that can create arbitrary culture medium formulations in 96 independent culture chambers has been developed (76). This microfluidic chip, which contained a number of pneumatic valves, was used to quantitatively analyze the influence of transient stimulations on the proliferation, osteogenic differentiation, and motility of human primary MSCs. Although these systems still lack the high-throughput capacity that is possible by miniaturization, they demonstrate the potential of fluidic systems for cell-based screening applications and high-throughput stem cell experimentations.

### 2.7. 3D Scaffolds for Controlling Stem Cell Differentiation 

**Figure 3.**
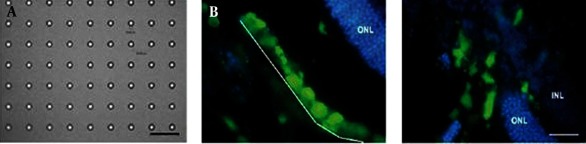
Retinal Progenitor Cells on Micro fabricated PMMA Scaffolds (87) A) Micro fabricated PMMA scaffold containing pores with 11 µm diameter and 6 µm depths. Scale bar is 100 µm; B) Retinal progenitor cells on a porous PMMA membrane. The Dashed white line indicates a PMMA membrane; C) Cells migrated into the photoreceptor (ONL) and inner nuclear layer (INL) of the host retina. Green and blue shows green fluorescent protein (GFP) cells and cell nuclei. Scale bar is 50 µm

The design of patterned scaffolds which possess the ability to differentiate adult stem cells into various lineages is a promising approach for controlling stem cell behaviors. Previously, a layer-by-layer laser micro fabrication approach has been used to create spatially patterned scaffolds of photocrosslinkable polymers (55, 56). This method can be used to pattern growth factors within 3D micro fabricated structures. Another study used soft lithography methods to fabricate biodegradable PLGA-based scaffolds (77). In this study, multi-layer structures were fabricated by thermally laminating each layer. Within those scaffolds, bone marrow cells differentiated into osteoblasts. To generate in vitro retinal tissues, poly(methyl methacrylate) (PMMA) scaffolds (11 µm diameter pores, 6 µm thick) were micro fabricated by using photolithography and reactive ion etching techniques and seeded with retinal progenitor cells treated with poly-L-lysine and laminin (77). Cells that attached to porous PMMA scaffolds migrated into host retinal layers after subretinal transplantation (Figure 3) (77). Cells expressing a neuronal marker (i.e. neurofilament-200) also extended through pores on PMMA scaffolds. Therefore, this micro fabricated PMMA scaffold could be useful for generating cytoarchitectural environment and can be potentially transplanted into the injured eye, because it increases cell survival and migration to specific retinal regions. Moreover, micro fabricated poly (glycerol-sebacate) (PGS) scaffolds with similar mechanical properties of retinal tissues have also been developed (78). To fabricate PGS scaffolds with 50 µm pores, the PDMS mold embossed with micro patterns was spin-coated with an aqueous sucrose layer. Molten PGS was subsequently spin-coated onto the sucrose-coated mold. After incubation, the PGS layer that was peeled off from the mold was treated by laminin to improve cell adhesion. Immunohistochemical analysis revealed that retinal progenitor cells cultured on porous PGS scaffolds for 7 days in vitro expressed neuronal markers. In addition to micro fabricated scaffolds, 3D scaffolds that can control mechanical stimulation and contain vascularized structures have been used to study stem cell behavior and to generate tissues by inducing their differentiation within the scaffold. In one example, oligo(poly(ethylene glycol) fumarate) (OPF) hydrogel scaffolds were used to differentiate and mineralize rat bone marrow stromal cells (79). Biomimetic OPF hydrogels were modified with Arg-Gly-Asp (RGD) peptides, which increased the numbers of differentiated cells cultured on the scaffold. In another study, porous PLGA scaffolds containing osteogenic factors (i.e. dexamethasone, ascorbate-2-phosphate) were used to investigate osteogenesis of MSCs (80). Ascrobate-2-phosphate enhanced the release rate of dexamethasone by increasing water uptake. It was demonstrated that MSCs cultured in PLGA scaffolds increased the amount of mineralization and induced osteogenic differentiation. Poly (caprolactone) (PCL) scaffolds fabricated by electrospinning were also used to examine the differentiation of bone marrow-derived human MSCs (81). When induced by specific differentiation media, human MSCs cultured in 3D nanofibrous PCL scaffolds exhibited adipogenic, chondrogenic, and osteogenic differentiation. Cells induced by TGF-β1 showed chondrocyte-like morphologies, while cells cultured in the presence of osteogenic supplements showed osteocyte-like morphologies. Scaffolds with controllable mechanical properties can be used to manipulate the microenvironment of adult stem cells (82) and direct stem cell differentiation. For example, the response of MSCs on hydrogel matrices that mimic tissue-level elasticity was analyzed to demonstrate that soft gel matrices (with stiffness of 0.1-1 kPa) generated neurogenic lineages, stiffer matrices (8-17 kPa) induced myogenic lineages, and rigid matrices (25-40 kPa) resulted in osteogenic lineages (83). In another study, cyclic compressive loading was used to induce chondrogenesis of bone-marrow MSCs in agarose constructs (84). Specimens were subjected to sinusoidal loading with 15% strain and cyclic compressive loading, which stimulated TGF-β signal transduction at the early stage of chondrogenesis. This dynamic compressive loading system could be useful for investigating mechanotransduction pathways in bone-marrow MSCs. As mentioned previously, engineered vascularized scaffolds play an important role in regenerating tissues and organs. Bone marrow cells have been manipulated by engineering approaches for generating vascularized structures. For example, decellularized matrices could be ideal scaffolds for regenerating vascular tissues due to their low immunogenicity in vivo, and favorable mechanical properties. Decellularized scaffolds have been used to generate vascular structures by seeding bone marrow-derived endothelial progenitor cells (EPCs) on scaffolds that were connected to recirculating perfusion systems (85). The immunohistochemistry and western blot analysis demonstrated the differentiation of the seeded EPCs into endothelial cells. It has been also demonstrated that EPCs can be purified from peripheral blood samples and subsequently combined with a polymeric scaffolding material (an acellular pig vessel) to form a tissue engineered blood vessel for implantation to treat vascular disorder (86).

## 3. Results

Since these cells potentially have the ability to regenerate the smooth muscle cells of the vessel, they may be useful in various clinical applications. Overall, it appears that micro engineered scaffolds can be used to provide a 3D microenvironment for adult stem cells. In particular, the 3D microarchitecture of the stem cell niche in the body provides biological necessity to use scaffolds. The future of this research seems promising in generating micro scale scaffolds that can be used to study biological mechanisms of cell response as well as therapeutic tissue engineered constructs.

## 4. Conclusions

Stem cells have enormous potential for therapeutic and pharmaceutical applications, because they can give rise to various cell types. Despite their therapeutic potential, many challenges, including the lack of control of the stem cell microenvironment remain. Thus, a greater understanding of stem cell biology that can be used to expand and differentiate embryonic and adult stem cells in a directed manner offers great potential for tissue repair and regenerative medicine. These challenges may be addressed by using micro engineering approaches such as surface patterning, high-throughput microarrays, microfluidics, and 3D biodegradable scaffolds (Table 1). As it can be seen by the increasing number of examples in the application of micro scale techniques for stem cell bioengineering, many advances have already been made, however, much progress still remains to be made to fully utilize these technologies.

**Table 1.** Micro Fabrication-based Techniques for Directing Stem Cell Fates

Micro engineering Approach	Technique	Biomaterial	Cell type	Study	Ref
**Surface pattern**	Stencil printing	Parylene-C	Murine ES ^a^ cell	Co-culture, Differentiation	(34, 42, 43)
	Microcontact printing	PDMS^a^, Matrigel	Human ES cell, Human MSC ^a^	Self-renewal, differentiation	(35, 68)
	Microwell	PEG ^a^, polyurethane	Murine ES cell Human ES cell	Homogeneous EB ^a^ size, Cell-cell contact, Self-renewal, Co-culture	(36, 37, 39-41)
	Layer-by-layer deposition	Hyaluronic acid, Poly-L-lysine	ES cell	Cell-cell contact	(38)
**Micro-array**	Robotic spotter	24 polymers	Human ES cell	Stem cell-biomaterial interaction	(44)
	Robotic spotter	Collagen I, III, IV Laminin, Fibronectin	Murine ES cell	Hepatic differentiation	(45)
	Robotic spotter	24 polymers	Human MSC	High-throughput screening	(69)
	Grid-based platform	Alginate, Collagen, Fibrin, Hyaluronic acid, Synthetic polymers	Human MSC, Human preadipocyte Human dental pulp stem cell, Murine ES cell, hematopoietic stem cell	Proliferation, apoptosis	(70)
**Micro-fluidic**	Reversibly sealing	PDMS	Murine ES cell	Cell-soluble factor interaction	(47)
	Multilayer channels	PDMS	ES cell	Homogeneous EB size	(48)
	Gradient-generator	PDMS	Human neural stem cell	Proliferation, Astrocyte differentiation	(71)
	Microfabricated platform	PDMS	Adult hippocampal progenitor	Cell tracking, Neuronal differentiation	(75)
Pneumatic valve, Peristaltic pump	PDMS	Human MSC	Osteogenic differentiation	(76)
**Scaffold**	Layer-by-layer stereo lithography	PEG, RGD ^a^	Murine marrow stromal cell, Murine MSC	Cell attachment, Osteogenicdifferentiation	(55, 56)
	Soft lithography	PLGA ^a^	Bone marrow cell	Osteogenic differentiation	(87)
	Photoligthography	PMMA ^a^	Retinal progenitor cell	Migration	(77)
	Soft lithography	PGS ^a^, PDMS	Retinal progenitor cell	Neuronal differentiation	(78)

^a^Abbreviation: EB, embryonic bodies; MSC, mesenchymal stem cells; PDMS, poly (dimethylsiloxane); PEG, poly (ethylene glycol); PGS, poly (glycerol-sebacate); PLGA, poly (lactic-co-glycolic acid); PMMA, poly (methyl methacrylate)
